# Assessment of reproductive performance in dairy cows using explainable machine learning

**DOI:** 10.5194/aab-69-469-2026

**Published:** 2026-08-28

**Authors:** Elif Çelik Gürbulak, Uğur Kara, Esra Canooğlu, Hazal Aysın Yüceel, Ece Çetin, Mehmet Demirel, Kutlay Gürbulak

**Affiliations:** 1 Department of Biometrics, Faculty of Veterinary Medicine, Erciyes University, Kayseri, Türkiye; 2 Department of Obstetrics and Gynecology, Faculty of Veterinary Medicine, Çukurova University, Adana, Türkiye; 3 Department of Obstetrics and Gynecology, Faculty of Veterinary Medicine, Erciyes University, Kayseri, Türkiye; 4 Department of Animal Science, Institute of Health Sciences, Erciyes University, Kayseri, Türkiye; 5 Boğazlıyan Vocational School, Yozgat Bozok University, Yozgat, Türkiye

## Abstract

Reproductive performance is a key determinant of productivity and economic sustainability in dairy farming and is influenced by a complex interaction of biological and management-related factors. This study aimed to evaluate reproductive performance in dairy cows and to identify the most influential risk factors at both global and individual animal levels using an explainable machine learning framework. An eXtreme Gradient Boosting (XGBoost) regression model was applied to evaluate reproductive performance based on days to first insemination. Model performance was assessed using standard regression metrics. Model interpretability was achieved through SHapley Additive exPlanations (SHAP), allowing both global feature importance assessment and local, animal-specific interpretations. SHAP analysis indicated that age had the greatest contribution to the model predictions, followed by mastitis, retained placenta, ovarian cysts, ketosis, and metritis. The direction and magnitude of the SHAP contributions varied across individual animals, highlighting heterogeneity in the model explanations. Model performance on the independent test dataset (RMSE = 39.05 d, MAE = 18.58 d, $R^{2}$ = 0.01) indicated limited predictive generalizability. Nevertheless, SHAP analysis provided transparent global and local explanations of model behavior, demonstrating how explainable machine learning can be applied to investigate reproductive performance in dairy cows. These findings should be regarded as a methodological demonstration, and further studies using larger, more comprehensive datasets that include a broader range of variables are required before clinical decision-support applications can be considered.

## 1 Introduction

Reproductive efficiency is one of the most important profitability parameters in dairy production systems. In dairy cows, reproductive performance is influenced by a wide range of physiological, pathological, and management-related factors. Epidemiological studies have reported that retained placenta, metritis, and ovarian cysts have direct adverse effects on reproductive performance (Fourichon et al., 2000). Metritis, particularly puerperal metritis, has been shown to reduce conception rates and prolong the interval to subsequent pregnancy (Giuliodori et al., 2013). In addition, the long-term negative effects of subclinical ketosis on reproductive efficiency are well documented, with affected cows exhibiting prolonged intervals from calving to first estrus, from calving to first artificial insemination, and from calving to conception (Rutherford et al., 2016).

Ovarian cysts in dairy cows represent another major cause of economic loss, as they are associated with extended calving-to-conception intervals, increased veterinary treatment costs, chronic infertility in some cases, and premature culling from the herd (Karsavuranoğlu et al., 2022). Moreover, recent studies have demonstrated that the effects of mastitis are not limited to the mammary gland but also extend to the reproductive system, leading to impaired fertility (Kumar et al., 2017). The decline in reproductive performance observed in cows with mastitis has been attributed to several mechanisms, including alterations in the hormonal profile, reduced oocyte quality, fertilization failure, an unfavorable uterine environment, and disturbances in early embryonic development. In addition, endotoxins produced by mastitis-causing pathogens are known to stimulate prostaglandin F2 α (PGF2 α ) release, thereby inducing luteolysis, altering the duration of the estrous cycle, and potentially causing embryonic or fetal losses (Mateus et al., 2003).

In veterinary medicine, numerous predictive models and analytical approaches have been developed to evaluate diseases, productivity, and performance traits, with the aim of improving preventive herd health management and reducing treatment-related costs. However, the high predictive performance of many machine learning (ML) models is often accompanied by “black-box” characteristics that obscure their decision-making processes. In recent years, the increasing complexity of artificial intelligence and ML models has reduced transparency and raised concerns regarding trust and interpretability, thereby accelerating the development of explainable artificial intelligence (XAI). XAI, also referred to as interpretable machine learning, seeks to elucidate the internal decision-making mechanisms of ML models and to identify the key features driving their predictions. Model interpretability can be achieved either by using inherently interpretable models, such as linear regression, logistic regression, and decision trees, or by applying post hoc explainability techniques to black-box models, including deep neural networks, random forests, eXtreme Gradient Boosting (XGBoost), and gradient boosting machines. An important advantage of post hoc methods is that they are model-agnostic and can be applied to both interpretable and black-box models (Lundberg et al., 2020; Angelov et al., 2021).

One of the most powerful XAI approaches is SHapley Additive exPlanations (SHAP), which quantifies the marginal contribution of each predictor to the model output for individual observations based on principles derived from game theory. SHAP is a feature-based interpretability method capable of explaining model behavior at both local and global levels and has become one of the most robust tools for model interpretation due to its strong theoretical foundation and its ability to quantify individual feature contributions (Angelov et al., 2021). In the SHAP framework, each independent variable is treated as a “player” in a cooperative game and receives a payoff proportional to its contribution to the prediction. SHAP is particularly efficient and computationally fast for tree-based models (Lundberg et al., 2020). The game-theoretic mathematical formulation of the SHAP method is given by

1 $$\varphi_{i}=\sum_{S\subseteq F\backslash \left\{i\right\}}\frac{\left|S\right|!\left(\left|F\right|-\left|S\right|-1\right)!}{\left|F\right|!}\left[f\left(S\cup \left\{i\right\}\right)-f(S)\right],$$

where $f\left(S\right)$ denotes the model output when the feature subset S is used; f(∅) denotes the baseline output of the model when no features are included; and f(F) denotes the model output when all features are included, $f\left(F\right)=f\left(\emptyset \right)+\sum_{i\in F}\varphi_{i}$ . (2) Let n denote the number of observations and $\varphi_{i}^{\left(k\right)}$ denote the SHAP value of the i th feature for the k th observation. The global importance of the i th feature is calculated as

2 $${\mathrm{importance}}_{i}=\frac{1}{n}\sum_{k=1}^{n}\left|\varphi_{i}^{\left(k\right)}\right|(3)$$

(Angelov et al., 2021, Lundberg et al., 2020).

Several studies published in the field of animal science have employed the SHAP methodology, increasingly emphasizing key considerations regarding its practical implementation. Chompo et al. (2026) applied support vector machines, random forest, XGBoost, and ridge logistic regression to predict *Opisthorchis viverrini* infection in cats using clinical, environmental, and management-related variables. Using SHAP analysis, they identified residence in flood-prone areas, feeding cats with fish remnants, and annual rainfall as the variables contributing most strongly to infection risk. Burton et al. (2024) developed a multimodal framework that integrated textual and tabular data from veterinary electronic health records in the United Kingdom to predict animal mortality, employing PetBERT, BERT-base, and tabular models for classification. Through SHAP analysis, they revealed that specific expressions in clinical narrative texts, such as descriptions of acute symptoms, had a stronger influence on model predictions than tabular variables, while age, breed, and socioeconomic scores also played significant roles in determining mortality risk. Cetintav and Yalçın (2025) investigated survival probability in horses with colic using random forest and XGBoost models based on clinical, laboratory, and lesion characteristics. By applying SHAP analysis, they examined both global and local model explanations and identified heart rate, lesion type, and total protein concentration as the most important determinants of survival.

In numerous studies comparing traditional statistical models with explainable artificial intelligence approaches, the advantages of the SHAP methodology have become increasingly evident. Zhou et al. (2026) used logistic regression together with random forest and gradient boosting models to predict mastitis and metabolic diseases in dairy cows and interpreted their results using SHAP. Similarly, Guo et al. (2025) compared logistic regression and XGBoost, along with several other machine learning algorithms, for predicting subclinical mastitis in dairy cows and employed SHAP analysis to evaluate the contribution and direction of time series milk production and composition features to XGBoost predictions. In the present study, the aim was to identify the factors influencing reproductive performance using the XGBoost algorithm in dairy cows by means of SHAP analysis.

## 2 Materials and methods

In this study, 466 observational records obtained from a dairy cattle farm were used to evaluate factors affecting reproductive performance. The dataset included information on age; days to first insemination; and disease indicators for metritis, mastitis, ketosis, retained placenta, and ovarian cysts for each animal. Each disease variable was coded as a binary indicator (0 = absent, 1 = present). In addition, 10 heifer observations were excluded from the dataset to ensure a homogeneous population consisting only of cows with complete reproductive histories. Prior to analysis, records with missing values were removed using a complete-case approach. Following repeated analyses, 17 observations with missing values were excluded from the initial dataset ( N = 456), resulting in a final sample size of N = 439 used in the analysis.

Reproductive performance was based on the number of days to first insemination. Fewer days to first insemination indicated better reproductive performance. XGBoost regression was employed to predict the reproductive performance and to model the effects of the independent variables. XGBoost is an ensemble-based machine learning method that demonstrates strong performance in capturing nonlinear relationships and interactions among predictors. The reproductive performance predicted by the model was categorized into three performance classes – low (0–33rd percentile), medium (34–66th percentile), and high (67–100th percentile) – based on percentile thresholds. Within each performance class, disease prevalence, mean age, and mean days to first insemination were calculated.

Hyperparameter tuning was performed on the training dataset using a grid search strategy combined with 5-fold cross-validation. A total of 432 parameter combinations were evaluated across maximum depth, learning rate (eta), subsample, colsample_bytree, and min_child_weight. The optimal parameter combination was identified as maximum depth = 2, eta = 0.05, subsample rate = 0.7, column subsampling rate per tree = 0.7, and minimum child weight = 3, with 100 boosting rounds and the lowest cross-validated RMSE (0.508). The final model was trained using a square error loss function (objective = reg:squarederror) and evaluated using root mean square error (RMSE), mean absolute error (MAE), and the coefficient of determination ( $R^{2}$ ).

To interpret the model's decision-making process and to quantify the contributions of individual predictors to reproductive performance, SHAP analysis was applied. Although the exact computation of Shapley values is nondeterministic polynomial-time hard (NP-hard), SHAP values in this study were obtained using the TreeSHAP algorithm implemented in XGBoost. TreeSHAP efficiently computes Shapley values by recursively propagating expected values through decision trees, reducing computational complexity from exponential to polynomial time (Lundberg and Lee, 2017; Lundberg et al., 2020). Both global- and local-level explanations were generated. Global feature importance was determined using the mean absolute SHAP values (mean | SHAP | ), thereby identifying the predictors most strongly influencing the model. In addition, variable effects were visualized using colored beeswarm plots, feature-wise scatter plots, and radar charts.

All data processing, analyses, and visualizations were performed using R software (version 4.5.0; R Core Team, 2025). The *dplyr* and *tidyr* packages were used for data manipulation, XGBoost for model fitting, *fastshap* and *shapviz* for SHAP analyses, *ggplot2 *and *viridis* for graphical visualization, and *fmsb* for radar charts. All analytical outputs were automatically saved to a predefined directory.

## 3 Results

In this study, the interpretability of the reproductive performance generated using the XGBoost regression model was evaluated through SHAP analyses. The model performance was assessed separately for the training and test datasets. The training dataset yielded RMSE = 19.79, MAE = 13.99, and $R^{2}$ = 0.18, while the test dataset yielded RMSE = 39.05, MAE = 18.58, and $R^{2}$ = 0.01 (Table 1).

**Table 1:** Model performance metrics for reproductive performance prediction.

Dataset	RMSE	MAE	$R^{2}$
Training	19.79	13.99	0.18
Test	39.05	18.58	0.01

* RMSE: root mean square error; MAE: mean absolute error; $R^{2}$ : coefficient of determination.

Based on the mean absolute SHAP values (mean | SHAP | ), age exhibited the highest importance among the predictors included in the model (mean | SHAP | = 3.205). Other variables making significant contributions to the model, in decreasing order of importance, were mastitis, retained placenta, ovarian cysts, ketosis, and metritis (Table 2).

**Table 2:** Global feature importance based on mean absolute SHAP values.

Feature	Mean | SHAP |
Age	3.205
Mastitis	0.324
Retained placenta	0.173
Ovarian cysts	0.079
Ketosis	0.073
Metritis	0.072

According to the XGBoost feature importance analysis, age emerged as the most dominant predictor, exhibiting the highest gain (gain = 0.892), covering the largest proportion of observations within the model (cover = 0.596), and being the most frequently used variable across decision trees (frequency = 0.633) (Table 3).

**Table 3:** Feature importance metrics of the XGBoost model.

Feature	Gain	Cover	Frequency
Age	0.892	0.596	0.633
Mastitis	0.044	0.086	0.095
Retained placenta	0.023	0.097	0.095
Ovarian cysts	0.019	0.093	0.078
Ketosis	0.013	0.072	0.053
Metritis	0.009	0.057	0.046

The reproductive performance was categorized into three classes – low, medium, and high – based on percentile thresholds. The distribution of animals across the low, medium, and high-performance classes was balanced, with $n_{1}$ = 149, $n_{2}$ = 143, and $n_{3}$ = 147, respectively. According to Table 4, disease prevalence differed markedly among the performance classes. Mastitis prevalence increased progressively from the high-performance group (19.6 %) to the low-performance group (38.4 %). Retained placenta was observed at a higher rate in the low-performance group (21.2 %). The interval from calving to first insemination was 56.27 d in the high-performance group and extended to 78.47 d in the low-performance group.

When evaluated by reproductive performance classes, metritis prevalence of the high-performance group ( n = 149) was 92.1 %, with a low prevalence of mastitis (19.6 %) and a high prevalence of ketosis (2.6 %). The mean interval to first insemination was 56.27 ± 7.31 d.

In the medium-performance group ( n = 143), metritis prevalence was 91.3 %, while mastitis prevalence was 34.0 %. Ketosis prevalence in this group was 2.7 %. The mean interval to first insemination was 63.47 ± 17.02 d.

In the low-performance group ( n = 149), a higher prevalence of mastitis (38.4 %) and retained placenta (21.2 %) were observed, along with metritis (94.6 %) and ketosis (1.4 %). Animals in this group exhibited a mean interval to first insemination of 78.47 ± 36.56 d (Table 4).

**Table 4:** Disease prevalence and reproductive indicators according to reproductive performance classes.

Reproductive		Metritis	Mastitis	Ketosis	Ovarian	RP	Mean
performance	n	rate (%)	rate (%)	rate (%)	cyst rate (%)	rate (%)	days to AI
Low	149	94.6	38.4	1.4	2.6	21.2	78.47 ± 36.56
Medium	143	91.3	34.0	2.7	5.3	17.3	63.47 ± 17.02
High	147	92.1	19.6	2.6	0.0	14.2	56.27 ± 7.31
Total	439	92.7	30.7	2.2	2.7	17.6	66.14 ± 25.44

* RP: retained placenta; AI: artificial insemination; NA: not available.

In the 10 highest-performing animals, disease prevalence and days to first insemination were generally lower, whereas higher values were observed in the 10 lowest-performing animals (Table 5).

**Table 5:** The 10 animals with the lowest and highest reproductive performance.

	Days to						
	first						
Animal	insemination		Retained				Ovarian
no.	postpartum	Age	placenta	Metritis	Mastitis	Ketosis	cysts
10 highest-performing animals							
264	31	68.8	0	1	1	0	0
248	32	69.8	0	1	0	0	0
355	34	44.7	0	1	0	0	0
379	34	40.2	0	1	0	0	0
278	35	72.7	1	1	0	0	0
43	37	72.9	0	1	1	0	0
254	37	69.5	0	0	1	0	0
369	37	48.2	0	0	1	0	0
282	38	71.7	1	1	0	0	0
335	39	48.4	0	1	0	0	0
10 lowest-performing animals							
314	318	51.3	0	1	0	0	0
47	258	34.6	0	1	0	0	0
440	184	37.8	0	1	1	0	0
30	160	38.1	0	1	0	0	0
455	158	35.3	0	1	0	0	0
3	153	35	0	1	0	0	0
452	151	38.2	0	1	0	0	0
463	143	33.1	1	1	0	0	0
444	139	34.8	0	1	1	0	0
69	133	34	0	1	0	0	0

* 0: absent; 1: present.

**Figure 1:**
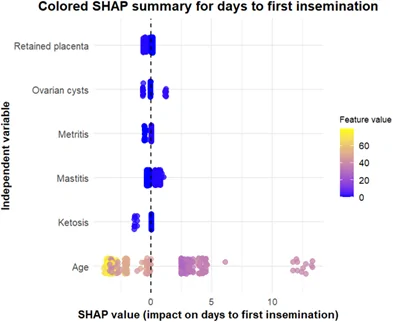
Distribution of SHAP values for the reproductive performance.

**Figure 2:**
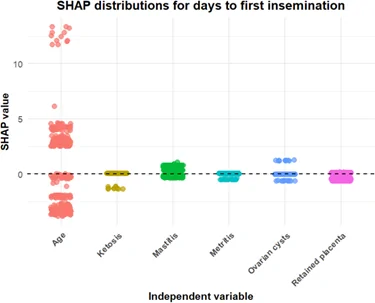
SHAP distributions associated with the reproductive performance.

The colored SHAP summary plot and the SHAP distribution plot indicated that age had the greatest contribution to the model predictions of days to first insemination. The progressive increase in positive SHAP values with increasing age indicates that older animals contributed more strongly to model predictions of longer intervals to first insemination. The predominance of positive SHAP values for postpartum diseases such as metritis, mastitis, retained placenta, and ovarian cysts suggests that these variables contributed positively to model predictions of longer intervals to first insemination. Ketosis also showed positive SHAP contributions; however, its relative contribution to the model predictions was lower than that of the other variables (Figs. 1–2).

**Figure 3:**
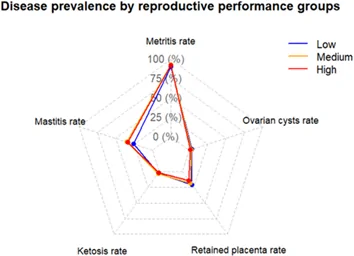
Radar chart of reproductive performance indicators for low-, medium-, and high-performance groups.

According to the radar chart results, animals in the low-reproductive-performance class exhibited a higher prevalence of metritis, mastitis, ketosis, and retained placenta compared with the other groups. In contrast, animals in the high-reproductive-performance group demonstrated markedly lower values in terms of disease prevalence. In the medium-reproductive-performance group, an increase in overall disease burden was associated with a pronounced decline in the reproductive performance (Fig. 3).

## 4 Discussion

In the dairy industry, the primary economic objective is to achieve high pregnancy rates alongside optimal milk yield. Milk production in dairy cows is directly linked to reproductive efficiency. Breeding heifers at the earliest appropriate age without compromising physiological and morphological development, initiating lactation early, maintaining annual calving thereafter, and preserving long-term breeding potential are fundamental goals of dairy herd management (Alpan and Arpacık, 1998; Taşkın et al., 2011). In addition to milk yield, numerous factors are known to influence conception rates in dairy herds (Lucy, 2001).

Silva et al. (1992) reported that parity, season, and year significantly affect the calving-to-first-insemination interval, the first-insemination-to-conception interval, the service period, and the calving interval. Pelister et al. (2000) reported a mean age at first calving of 30 months in Holstein cows in the Marmara region and identified year and season as significant sources of variation, with year exerting the greatest effect on reproductive traits. In another study, the mean number of inseminations per conception was reported as 2.4, reaching its highest values at the sixth and seventh pregnancies. Kaya and Bardakçıoğlu (2016) demonstrated that herd and age at calving had statistically significant effects on age at first pregnancy, while age and year at calving significantly influenced age at first insemination. Sung et al. (2016) reported significant differences in the second calving interval among early- ( <24 months), moderate- (24–28 months), and late-age-at-first-calving ( >28 months) groups.

In the present study, age had the highest mean absolute SHAP value (3.205) among the predictors included in the fitted model. Within the fitted model, increasing age contributed more strongly to model predictions of longer intervals to first insemination, whereas younger animals were predicted to have shorter intervals to first insemination (Table 3). Consistent with these findings, Cielava et al. (2017) reported a decrease in conception probability with increasing parity, while other studies have shown that pregnancy rates remain relatively high up to the fourth lactation but decline thereafter.

An increased incidence of diseases such as mastitis in cows affected by peripartum metabolic disorders is considered a major contributor to reduced fertility (Bruinjé and LeBlanc, 2025). Previous studies have demonstrated that clinical mastitis is associated with lower conception rates and an increased number of inseminations per pregnancy. Mastitis complicates estrus detection, prolongs the calving-to-conception interval, induces anovulatory cycles, and reduces oocyte quality and fertilization rates. Compared with clinical mastitis, subclinical mastitis has been reported to exert a relatively milder negative impact on fertility (Hansen et al., 2004; Dolecheck et al., 2019). In the present study, mastitis had the second-highest mean absolute SHAP value among the predictors included in the fitted model (0.324). Mastitis prevalence increased progressively from the high- to the low-performance class (Table 4).

Impairment of smooth muscle function and its adverse effects on dystocia, retained placenta, uterine involution, and neutrophil function are well documented and are expected to indirectly compromise reproductive function. In agreement with this mechanism, hypocalcemic cows have been reported to experience delayed resumption of postpartum cyclicity, prolonged postpartum anestrus, and up to a 50% reduction in first-service conception rates (Caixeta et al., 2017). Immunosuppressed cows are also at increased risk of metabolic disorders such as retained placenta (Roche, 2006). In the present study, retained placenta exhibited a mean SHAP value of 0.173, with its prevalence increasing from the high- to the low-performance group (Table 4).

Ovarian cysts showed a relatively smaller effect on reproductive performance (mean SHAP value = 0.079). The prevalence of ovarian cysts was higher in the medium-performance group than in the high-performance group. Consistent with these findings, previous studies have reported that ovarian cysts are commonly observed during the postpartum period in dairy cows and represent an important cause of infertility by prolonging the calving-to-conception interval (Borş and Borş, 2020; Karsavuranoğlu et al., 2022).

Ketosis has previously been shown to reduce both milk yield and reproductive performance while increasing the risk of early culling in affected cows (Vanholder et al., 2015). In the present study, ketosis ranked fifth among the factors influencing reproductive performance (mean SHAP value = 0.073), with the highest prevalence observed in the low-performance group (Table 4).

Sheldon et al. (2009) emphasized that the detrimental effects of metritis on fertility arise through delayed resumption of ovarian cyclicity, disruption of the uterine environment, and impaired embryo development. In the present study, metritis had the lowest mean absolute SHAP value among the predictors in the fitted model (0.072).

Within the fitted model, mastitis and retained placenta showed relatively larger SHAP contributions than the remaining disease variables (Table 2).

Zhou et al. (2026) demonstrated that SHAP analysis can complement conventional statistical approaches by revealing the direction and magnitude of individual feature contributions to machine learning predictions in dairy cows. In a comprehensive review, Shahid et al. (2025) emphasized that logistic regression is more suitable for causal interpretation, whereas SHAP is better suited for the clinical interpretation of complex models.

Lundberg and Lee (2017) theoretically compared SHAP with methods such as partial dependence plots (PDPs), LIME, DeepLIFT, and Shapley sampling, demonstrating its axiomatic superiority. They concluded that SHAP is the only additive feature attribution method that simultaneously satisfies local accuracy, consistency, and missingness. SHAP was shown to integrate the strengths of existing approaches by providing both local accuracy and global interpretability. Goldstein et al. (2015) noted that PDPs may be misleading in the presence of strong interactions, whereas individual conditional expectation (ICE) plots reveal observation-specific functional relationships. SHAP extends beyond these approaches by delivering both global and local contributions on a unified scale. Ribeiro et al. (2016) emphasized that local explanations are essential for assessing trust in individual predictions and that models should be compared not only based on predictive accuracy but also on the quality of their explanations. In the present study, SHAP effectively captured both global and local effects

Although SHAP analysis facilitated the interpretation of the relative contributions of the predictors learned by the XGBoost model, the model performance on the independent test dataset ( $R^{2}=0.01$ ) indicates that the current model has limited generalizability. Therefore, the SHAP importance values should be interpreted as explanations of the patterns learned by the fitted model rather than as definitive evidence of causal or universally generalizable biological relationships. The limited predictive accuracy suggests that important variables influencing reproductive performance may have been insufficiently represented in the available dataset or were not included in the model. Future studies should incorporate additional reproductive, nutritional, genetic, environmental, and management-related variables, as well as data from larger and more diverse dairy herds, to improve model robustness and external validity.

In conclusion, this study demonstrates the applicability of an explainable machine learning framework for investigating reproductive performance in dairy cows. SHAP analysis enabled transparent interpretation of the XGBoost model by providing both global- and individual-level explanations of predictor contributions. However, the low predictive performance observed in the independent test dataset ( $R^{2}=0.01$ ) indicates that the current model has limited generalizability and should not yet be considered suitable for clinical decision support or herd-level management. Rather, this study should be regarded as a methodological proof-of-concept illustrating the integration of XGBoost and SHAP in veterinary data analysis. Future studies based on larger datasets incorporating a broader range of variables are required to develop predictive models suitable for clinical applications.

## Data Availability

The data are not publicly available because they contain farm-specific information but are available from the corresponding author upon reasonable request.
